# 3-D spatial floating display using multi-wavelength integral photography

**DOI:** 10.1038/s41598-018-33730-2

**Published:** 2018-10-26

**Authors:** Zhencheng Fan, Yan Xia, Hongen Liao

**Affiliations:** Department of Biomedical Engineering, School of Medicine, Tsinghua University, Beijing, 100084 China

## Abstract

Three-dimensional (3-D) autostereoscopic display with dedicated multiple spatial information under corresponding illumination is critical, especially for anti-counterfeiting, entertainment, etc. In this paper, we propose a 3-D spatial floating display using multi-wavelength integral photography (IP). Using dedicated inkjet printer and refraction-based IP algorithm, a complex two-dimensional (2-D) elemental image array (EIA) can be printed for both fluorescent and normal 3-D autostereoscopic display. With a micro-convex lens array (MLA) and a medium attached on the EIA, normal 3-D images are reconstructed under visible light, while fluorescent 3-D images can be reconstructed under ultraviolet (UV) light. Moreover, to provide comfortable 3-D images with multiple information in space, a feasible 3-D spatial floating display system is also proposed considering the spatial position of the observer with less UV radiation. The proposed method takes the wavelength of 3-D display into consideration to provide spatial multi-information, and can be applied for media, entertainment, etc. Experimental results verified the availability of the proposed method.

## Introduction

Compared with conventional 2-D displays, 3-D displays attract lots of attention due to the displayed images with spatial information. As main components in 3-D displays, 3-D autostereoscopic displays, mainly consisting of parallax barrier-based display, lenticular-based display and integral photography (IP)-based display, holography, etc. can provide 3-D images without any supplementary glasses or tracking devices^1,2^. The parallax barrier-based as well as the lenticular-based 3-D autostereoscopic displays have compact system configuration and are capable of providing objects with lateral parallax^3^. The holography-based 3D autostereoscopic displays can display a fully 3-D image of the holographed subject based on conventional lens imaging^4^. The configuration of the holography system, including the hologram is complicated to fabricate for accurate 3-D display with abundant spatial information. As a promising 3-D autostereoscopic display technique, IP has a simplified configuration, and can display 3-D autostereoscopic images with full parallax that observers can view. Moreover, the spatial information of the 3-D images can be condensed using the micro-convex lens array (MLA) and recorded into the 2-D elementary image array (EIA)^5^. The 3-D image can be reconstructed in space by the reversibility of the light ray. Performance of the 3D autostereoscopic display, like the viewing angle, the visualization depth and the resolution, etc., was improved in current research^6^. For instance, curved lens arrays, dynamic barriers, adaptive liquid crystal prism arrays and other novel components have been proposed to improve the viewing angle limited by the field of view of the optical lens^7–9^. IP rendering algorithm including referential viewing area-based rendering algorithm, and novel optical setups, such as micro-electro-mechanical system (MEMS) are utilized to display 3-D image with long distance^10–13^. To improve the resolution of displayed 3-D images, rotated prism sheets, multi-projector, etc. are presented^14,15^.

With merits of displaying 3-D images, 3-D autostereoscopic display techniques have been applied widely. Moreover, 3-D autostereoscopic displays, which can provide different spatial information under illumination with multi-wavelength are of importance in various filed, like media, entertainment, etc.^16–18^. For instance, in entertainment application, individuals prefer to observe fancy and changeable 3-D autostereoscopic images under different environment, rather than conventional 3-D images with single mode.

In this paper, we take the wavelength of the illumination in 3-D display into consideration and present a 3-D spatial floating display using multi-wavelength IP, especially for entertainment. Using dedicated inkjet printer and refraction-based IP algorithm, complex 2-D EIA with both visible and invisible information can be printed for 3-D autostereoscopic display under illumination with multi-wavelength. A feasible 3-D spatial floating display system, consisting of a MLA, a medium and a movable half-silvered mirror is also proposed to avoid the injury caused by directly observing the UV light and provide comfortable 3-D floating images with multiple information in space considering the spatial position of the observer. Therefore, individuals can observe normal 3-D images under visible light, while 3-D fluorescent images under UV light. The proposed method can be applied for 3-D anti-counterfeit with complex spatial information and high security, media as well as entertainment. Experiments verified the availability of the proposed method and system.

## Multi-Wavelength Ip-Based 3-D Spatial Floating Display Method

**Figure 1:**
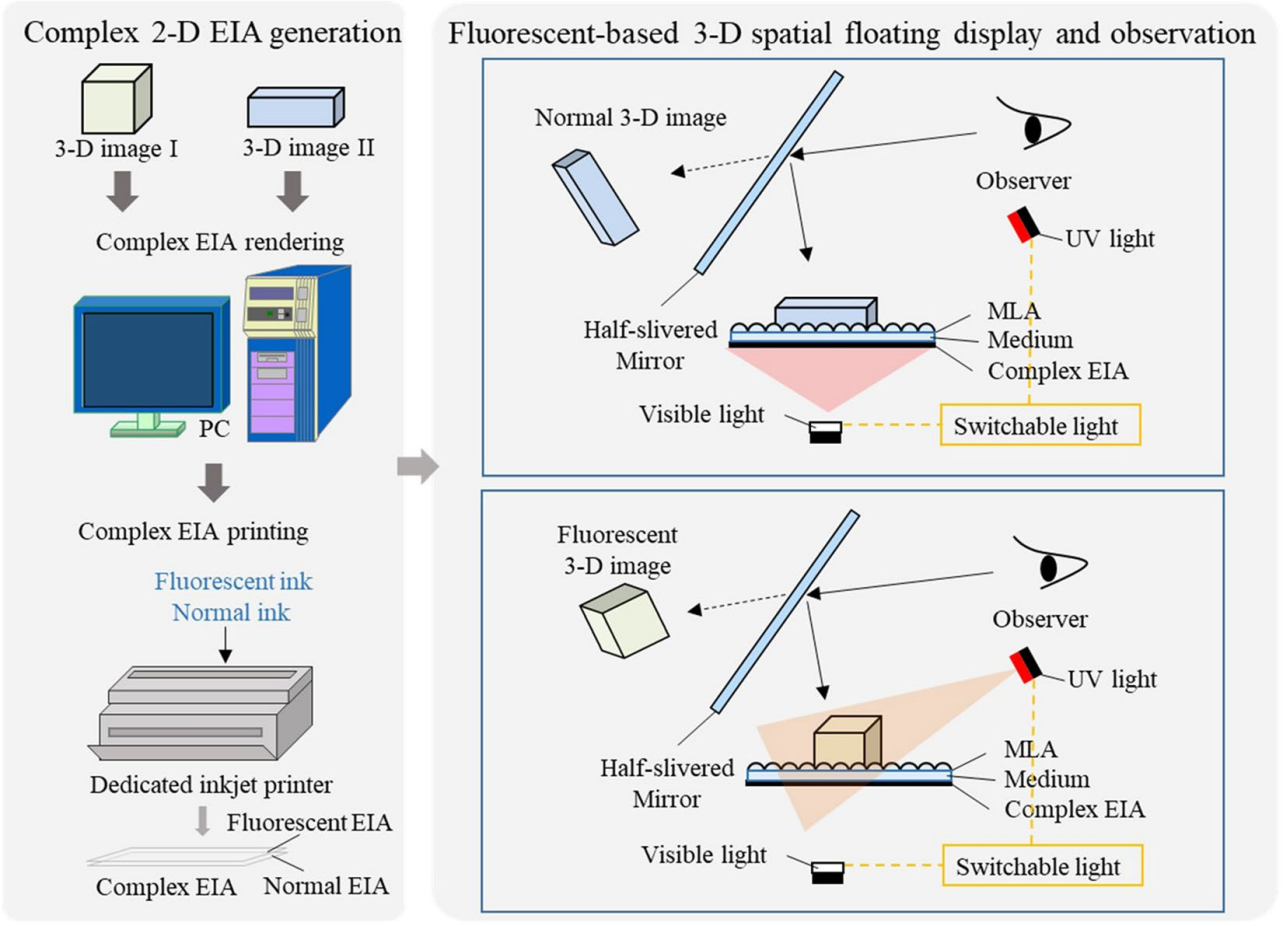
Process of the 3-D spatial floating display using fluorescent-based IP.

The proposed multi-wavelength IP-based 3-D spatial floating display method includes 2-D complex EIA generation and spatial floating display based on IP technique, as shown in Fig. 1. After designing required 3-D images, we proposed a refraction-based EIA rendering algorithm to render a complex EIA. The complex EIA consists of an EIA for 3-D fluorescent display and an EIA for normal 3-D display. Considering the printing mode of the dedicated inkjet printer, which is improved by a normal inkjet printer, the EIA for 3-D fluorescent display should be processed for half-toning printing. A dedicated 3-D spatial floating display system is used for providing the intuitive 3-D images in space. The printed complex EIA is arranged under a medium and a MLA with the corresponding parameters, while a flexible half-silvered mirror can be adjusted according to the position of the individual for better observation.

### Complex EIA generation

The 2-D complex EIA generation method can condense the designed 3-D spatial images into 2-D EIA, which will be printed on objects using a dedicated inkjet printer with both fluorescent ink and normal ink.

**Figure 2:**
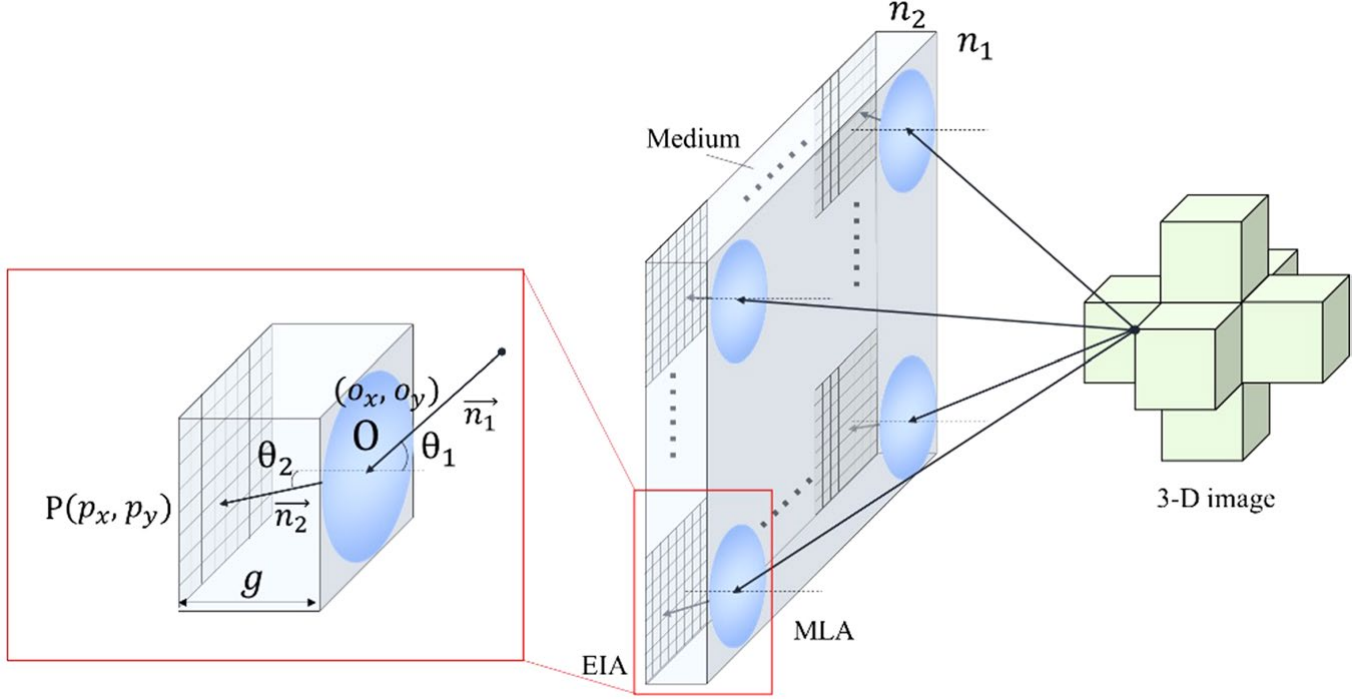
Principle of the refraction-based EIA rendering.

The complex EIA is arranged under a medium attached to the MLA in display process, thus, the thickness and the refraction index of the medium between the complex EIA and MLA should be taken into consideration in accurate EIA rendering^19^. To simplify the rendering model, each lens in MLA is considered as a pinhole model, and the detected object is attached closely to the medium. The refraction index of the MLA is almost identical to the one of the medium. The disparity of the refraction index between the MLA and the air brings out light refraction, as shown in Fig. 2. *n*_1_ is the refraction index of the air, while *n*_2_ is the refraction index of the medium. $\mathop{{n}_{1}}\limits^{ \rightharpoonup }$ represents the light ray emitting from the simulated 3-D image to the center of the corresponding lens in MLA, while $\mathop{{n}_{2}}\limits^{ \rightharpoonup }$ represents the light ray from the lens to the pixel in EIA. $\mathop{{n}_{2}}\limits^{ \rightharpoonup }$ is denoted by (*x*_*2*,_ *y*_*2*,_ *z*_*2*_), while $\mathop{{n}_{1}}\limits^{ \rightharpoonup }$ is denoted by (*x*_*1*,_ *y*_*1*,_ *z*_*1*_). The relationship between (*x*_2,_ *y*_*2*,_ *z*_*2*_) and (*x*_1,_ *y*_*1*,_ *z*_*1*_) can be represented by:

1 $$\begin{array}{rcl}{x}_{1} & = & {x}_{2}={p}_{x}-{o}_{x}\\ {y}_{1} & = & {y}_{2}={p}_{y}-{o}_{y}\\ {z}_{2} & = & g\\ {z}_{1} & = & \frac{{\Vert ({x}_{2},{y}_{2})\Vert }_{2}}{\tan (\arcsin (\frac{{n}_{2}}{{n}_{1}}\frac{{\Vert ({x}_{2},{y}_{2})\Vert }_{2}}{{\Vert ({x}_{2},{y}_{2},{z}_{2})\Vert }_{2}}))}\end{array}$$

where *g* is the thickness of the medium. When (*x*_2,_ *y*_*2*,_ *z*_*2*_) is confirmed in 3D rendering based on the position of rendered pixel and the position of the corresponding lens in MLA, (*x*_1,_ *y*_*1*,_ *z*_*1*_) can be calculated. Vectors of the incident light ray and outgoing light can be calculated for rendering an accurate EIA.

The complex EIA consists of an EIA for 3-D fluorescent display and an EIA for normal 3-D display. By replacing the ink in the cartridge of a normal inkjet printer by a fluorescent ink, the rendered EIA for 3-D fluorescent display can be printed to objects by the fluorescent inkjet printer. Moreover, considering different working modes of the fluorescent inkjet printer, the rendered EIA should be pre-processing for binary printing. To simplify the fluorescent printing, the recommend method is injecting the fluorescent ink into the cartridge for black-and-white printing, choosing the printing mode as black-and-white printing, and processing the rendered EIA into gray value. The normal EIA can be printed using the color-printing model. Therefore, observers can only view 3-D autostereoscopic images under visible illumination while view the hidden 3-D fluorescent images under UV light.

### 3-D spatial floating display

**Figure 3:**
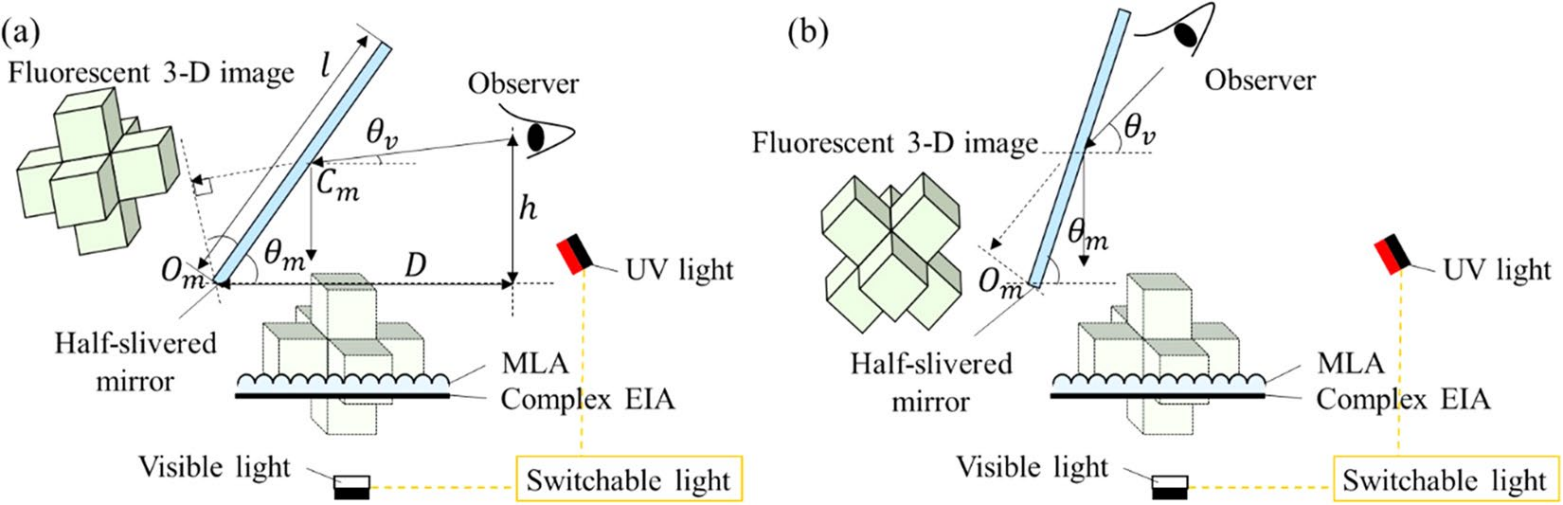
Flexible 3-D spatial floating display using multi-wavelength IP considering the observer.

Main components in the 3-D spatial floating display system are the switchable light including visible light and UV light, the MLA with corresponding parameters and the flexible half-slivered mirror. The MLA is utilized for reconstructing the light field information of the 3-D image. To avoid the injury caused by directly observing the UV light and provide floating 3D images, a half-silvered mirror is arranged above the MLA. To avoid the reversed image reconstructed by the half-silvered mirror, we should reverse the 3D model and render a correct EIA. Moreover, to provide intuitive 3-D images with multiple spatial information flexibly, we design a rotatable half-silvered mirror, which can be adjusted according to the viewing angle of the observer (Fig. 3). *O*_*m*_ is the fix position of the half-silvered mirror, *C*_*m*_ is the midpoint of the half-silvered mirror. The viewing angle *θ*_*v*_, the angle of the half-silvered mirror *θ*_*m*_, the observation distance *D* and the observation height *h* satisfy:

2 $$\begin{array}{rcl}{\theta }_{v} & = & 2{\theta }_{m}-90^\circ \,({\theta }_{m}\ge 45^\circ )\\ h & = & \tan \,{\theta }_{v}(D-0.5l\,\cos \,{\theta }_{m})+0.5l\,\sin \,{\theta }_{m}\end{array}$$

where *l* is the length of the half-silvered mirror. When the observation distance and the observation height are determined, the angle of the half-silvered mirror can be calculated for intuitive 3-D autostereoscopic display with multi-wavelength.

## Experiments and Results

**Figure 4:**
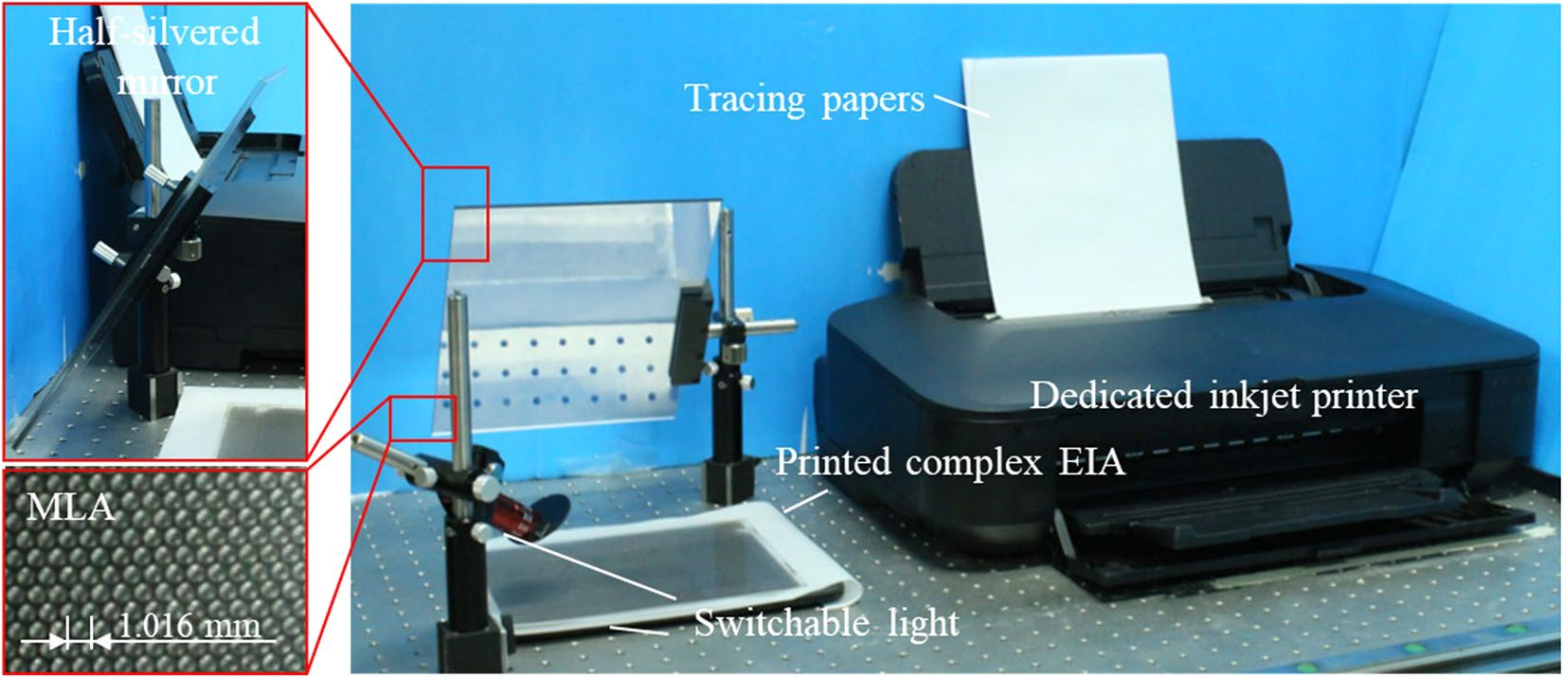
Fabricated 3-D spatial floating display using multi-wavelength IP.

We fabricated a 3-D spatial floating display system using multi-wavelength integral photography IP and rendered various 3-D images to operate experiments for verifying the feasibility of the proposed method, as shown in Fig. 4. The fabricated system consists of a switchable light including a UV light to inspire the fluorescence and a visible light, a MLA attached to a medium to construct the 3-D images, and a half-silvered mirror for intuitive spatial floating observation according to the observer’s position. Limited by the illumination setups, the light is switched manually. The wavelength of the UV light is 365 nm. To remove the effect of the visible light in the UV light, an optical filter plate is arranged over the UV light. The diameter of each lens in the hexagonal MLA is 1.016 mm, while the focal length is about 3.0 mm. The number of pixel in rendered EIA is 4500 × 4500. The number of lens is about 125 × 144. The thickness of the medium is around 3.0 mm. Moreover, we chose an inkjet printer (Canon ix6700), cleaned the cartridge and injected the fluorescent ink into the cartridge for black-and-white printing. The fluorescent ink can emit blue ray when lighten by UV light.

**Figure 5:**
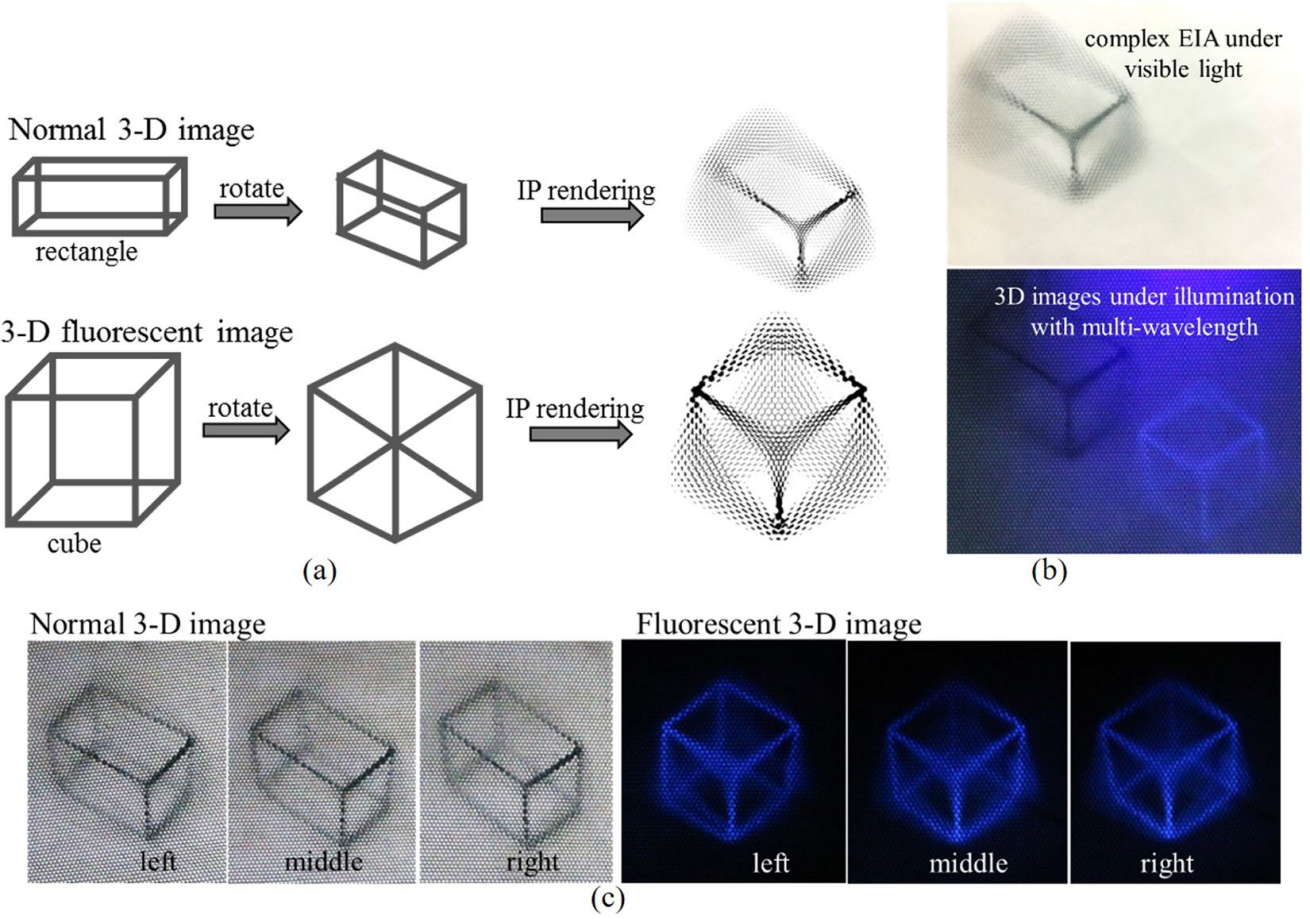
Experiments on 3-D spatial floating display system. (**a**) The designed 2-D complex image and the rendered EIA. (**b**) The printed complex EIA illuminated by visible light and reconstructed 3D images under illumination with multi-wavelength. (**c**) Observation results of the 3-D fluorescent autostereoscopic image under different illuminations from different aspects.

The 3-D fluorescent image is designed as a cube, while the normal 3-D image is designed as a rectangle (Fig. 5(a)). The complex EIA was rendered using refraction-based EIA rendering algorithm. The EIA for fluorescence-based 3-D display was printed by choosing the black-and-white printing model and the EIA for normal 3-D display was printed by the color-printing model. The complex EIA, including two EIAs was printed on the tracing papers separately using the dedicated inkjet printer at 900 pixels per inch (ppi), as shown in Fig. 5(b). The fluorescent EIA was aligned and arranged above the normal EIA. Under illumination with multi-wavelength, 3-D images including the normal 3-D image and the fluorescent 3-D image can be both observed. Individuals only observe the normal EIA under the visible light. When putting the EIA under the corresponding MLA, individuals can view the normal 3-D image under the visible light and the 3-D fluorescent autostereoscopic image for anti-counterfeiting or entertainment from different aspects under the UV light (Fig. 5(c)). Limited by the intensity of the UV light, some parts of the reconstructed 3-D image are not obvious because that without backlight, no images can be observed. Compared to the direct observation, the floating 3-D images have less intensity and contrast due to the half-silvered mirror.

## Discussion

To the best of our knowledge, this work is the first one to take the multi-wavelength of 3-D display into consideration and presents a 3-D spatial floating display using multi-wavelength IP. Considering the optical setups of the IP-based 3D image display, a refraction-based rendering algorithm is utilized for generating accurate complex EIA. The complex EIA is printed by a dedicated inkjet printer for both normal 3-D display and fluorescence-based 3-D display. To operate binary fluorescent printing, the conventional normal inkjet printer is improved and the fluorescent ink is injected into the corresponding cartridge of a normal inkjet printer. Moreover, error diffusion-based half-tone rendering algorithms can also be utilized according to demands^20^. With the proposed method, individuals can view normal 3-D image under visible light, while observe the 3-D fluorescent autostereoscopic image under UV light. Moreover, using the flexible 3-D spatial floating system, observer can view the floating 3-D autostereoscopic image with multi-wavelength in space intuitively.

Experiments verified the feasibility of the proposed method. Limited by the pixel density of EIA and the length pitch of each lens in MLA^6^, the reconstructed 3-D autostereoscopic images had a rough resolution. The size of the switchable light can influence the image quality because that no image can displayed without backlight. To improve the performance of the IP-based 3-D autostereoscopic image, high-quality MLAs or other specific optical setups could be taken into consideration to generate 3-D images with high quality, such as large viewing angle^21^, long display distance^22^ and high resolution. Moreover, the size and quality of the illumination could be improved as a uniform light source. In this paper, we simplify the model of the half-silvered mirror and regard there is only one reflection, and further study should be considered. Moreover, we utilize the tracing papers in the complex EIA printing. Other materials, which can absorb different types of ink and better transparency will be taken into consideration. Considering that different color could be inspired from various fluorescence with different properties, fluorescence with better performance^23,24^ and invisible fluorescent-based 3D image printing with multi-color will be researched in further study. Other light band will also be considered for multi-wavelength IP. Associated with other techniques, the proposed method can be applied in marker-based surgical navigation^25^. If the fluorescence can be controlled flexibly, dynamic 3-D fluorescence images will be reconstructed for interactivity^26,27^.

## Conclusion

In this paper, we proposed a 3-D spatial floating display using multi-wavelength IP. A complex EIA printing method, a refraction-based IP rendering algorithm and a flexible spatial floating display system are presented to provide intuitive 3-D images with multiple information in space. Normal 3-D images are reconstructed under visible light, while fluorescent 3-D autostereoscopic images can be reconstructed under UV light. The proposed method can be applied for specific 3-D display under different illumination, especially in entertainment. It also can be utilized in 3-D anti-counterfeit with complex spatial information and high security, media, etc.
